# A novel immune-related model to predict prognosis and responsiveness to checkpoint and angiogenesis blockade therapy in advanced renal cancer

**DOI:** 10.3389/fonc.2023.1127448

**Published:** 2023-03-14

**Authors:** Peng Chen, Feng Bi, Weili Tan, Lian Jian, Xiaoping Yu

**Affiliations:** Department of Diagnostic Radiology, Hunan Cancer Hospital and the Affiliated Cancer Hospital of Xiangya School of Medicine, Central South University, Changsha, Hunan, China

**Keywords:** renal cell carcinoma, IMmotion151, checkpoint blockade, antiangiogenesis, responsiveness, prognosis

## Abstract

**Background:**

Immune checkpoint blockade (ICB) and anti-angiogenic drug combination has prolonged the survival of patients with advanced renal cell carcinoma (RCC). However, not all patients receive clinical benefits from this intervention. In this study, we aimed to establish a promising immune-related prognostic model to stratify the patients responding to ICB and anti-angiogenic drug combination and facilitate the development of personalized therapies for patients with RCC.

**Materials and methods:**

Based on clinical annotations and RNA-sequencing (RNA-seq) data of 407 patients with advanced RCC from the IMmotion151 cohort, nine immune-associated differentially expressed genes (DEGs) between responders and non-responders to atezolizumab (anti-programmed death-ligand 1 antibody) plus bevacizumab (anti-vascular endothelial growth factor antibody) treatment were identified *via* weighted gene co-expression network analysis. We also conducted single-sample gene set enrichment analysis to develop a novel immune-related risk score (IRS) model and further estimate the prognosis of patients with RCC by predicting their sensitivity to chemotherapy and responsiveness to immunotherapy. IRS model was further validated using the JAVELIN Renal 101 cohort, the E-MTAB-3218 cohort, the IMvigor210 and GSE78220 cohort. Predictive significance of the IRS model for advanced RCC was assessed using receiver operating characteristic curves.

**Results:**

The IRS model was constructed using nine immune-associated DEGs: *SPINK5*, *SEMA3E*, *ROBO2*, *BMP5*, *ORM1*, *CRP*, *CTSE*, *PMCH* and *CCL3L1*. Advanced RCC patients with high IRS had a high risk of undesirable clinical outcomes (hazard ratio = 1.91; 95% confidence interval = 1.43–2.55; P < 0.0001). Transcriptome analysis revealed that the IRS-low group exhibited significantly high expression levels of CD8^+^ T effectors, antigen-processing machinery, and immune checkpoints, whereas the epithelial–mesenchymal transition pathway was enriched in the IRS-high group. IRS model effectively differentiated the responders from non-responders to ICB combined with angiogenesis blockade therapy or immunotherapy alone, with area under the curve values of 0.822 in the IMmotion151 cohort, 0.751 in the JAVELIN Renal 101 cohort, and 0.776 in the E-MTAB-3218 cohort.

**Conclusion:**

IRS model is a reliable and robust immune signature that can be used for patient selection to optimize the efficacy of ICB plus anti-angiogenic drug therapies in patients with advanced RCC.

## 1 Introduction

RCC is the 12^th^ most common solid tumor that accounted for > 400,000 new diagnoses and approximately 175,000 cancer-associated deaths worldwide in 2018 (1). Approximately 25% of RCC cases are diagnosed at an advanced stage (2). Clear cell RCC (ccRCC) is the most frequent histological subtype, accounting for approximately 75% of all renal tumors (3). Approximately 20% of patients with metastatic RCC (mRCC) have sarcomatoid elements. Sarcomatoid RCC (sRCC) is a rare subtype of RCC characterized by aggressive biology with rapid metastasis, unsatisfactory clinical outcomes, and limited efficacy of anti-angiogenic therapies (4–6).

Loss or mutation of the von Hippel-Lindau (*VHL*) gene is one of the primary characteristics of ccRCC that leads to the constitutive activation of the hypoxia-inducible factor, which further activates the vascular endothelial growth factor (VEGF) and increases angiogenesis in the ccRCC tumor microenvironment (7–12). Targeting the VEGF pathway with receptor tyrosine kinase inhibitors (TKIs), such as sunitinib, or anti-VEGF monoclonal antibodies, such as bevacizumab, is the first-line treatment for locally advanced or metastatic RCC (13, 14). However, almost all patients develop drug resistance over time, and particular patient subgroups, including those with sRCC and/or those expressing the programmed death-ligand 1 (PD-L1), hardly benefit from VEGF pathway blockade (15–17). Therefore, it is necessary to explore novel therapeutic targets and drug combinations for patients with mRCC (18, 19).

Intervention with immune checkpoint inhibitors (ICIs), such as anti-PD-L1 antibody atezolizumab, has induced durable responses and improved the survival of patients with mRCC (16, 20). T cell-mediated tumor cytotoxicity of atezolizumab can be strengthened by counteracting the VEGF-mediated immunosuppressive effect *via* the addition of bevacizumab (21). Owing to variable hypervascularity, immune cell infiltration, and PD-L1 expression in ccRCC, blocking the VEGF pathway and PD-L1 axis as a combination therapy has significantly prolonged the overall survival (OS) of patients with mRCC. A phase 2 study revealed that in a subset of patients with mRCC with PD-L1 expression, compared to sunitinib as a single drug, atezolizumab combined with bevacizumab significantly increased progression-free survival (PFS) and percentage of patients achieving an objective response, indicating the complementary activity of bevacizumab and atezolizumab in patients with mRCC (20). To reduce the financial burden and side effects of tumor therapy, it is necessary to develop effective strategies to select a subgroup of patients who can achieve optimal improvement with a specific combination therapy for mRCC.

In this study, we aimed to correlate the clinical annotation with molecular mechanisms by comprehensively analyzing the multi-omics information of 407 patients with advanced RCC from a randomized global phase III trial (IMmotion151). We also established a novel and promising prognostic model composed of nine immunotherapy-associated genes to accurately stratify a subset of patients with advanced RCC who can benefit from anti-angiogenic combined with ICB (atezolizumab plus bevacizumab) therapy. Moreover, our model can be used to develop personalized treatment strategies for patients with advanced RCC.

## 2 Materials and methods

### 2.1 Collection and processing of data

To determine the correlation between the immune-related risk score (IRS) and efficacy of cancer therapy, five immunotherapeutic cohorts with available RNA-seq data and clinicopathological parameters were included in this study: (1) IMmotion151 cohort, advanced patients with RCC treated with atezolizumab plus bevacizumab (22), (2) JAVELIN Renal 101 trial, advanced patients with RCC treated with the combination of avelumab (anti-PD-L1) + axitinib (TKI targeting VEGF receptors) vs. sunitinib (multitarget TKI) (23), (3) E-MTAB-3218 dataset, patients with mRCC treated with nivolumab (anti-PD-1 ICI) (24), (4) IMvigor210 cohort, patients with advanced urothelial cancer treated with atezolizumab (25), and (5) GSE78220 cohort, patients with metastatic melanoma treated with pembrolizumab (anti-PD-1 antibody) (26).

All data from the IMmotion151 cohort is deposited in the European Genome-Phenome Archive under the accession number EGAS00001004353, and we obtained it according to Hoffmann-La Roche policy. Clinical response information and normalized RNA-seq data were acquired from the supplementary material of Choueiri et al. (23). Original data of E-MTAB-3218 dataset were downloaded from https://www.ebi.ac.uk/biostudies/arrayexpress/studies/E-MTAB-3218?accession=E-MTAB-3218# (24). Additionally, RNA-seq and clinical data for the IMvigor210 cohort were obtained from http://research-pub.gene.com/IMvigor210CoreBiologies. Raw data were normalized using the “DEseq2” R package and further transformed into TPM values. RNA-seq data (FPKM-normalized) and the clinical phenotypes of 28 melanoma patients in the GSE78220 cohort were downloaded from https://www.ncbi.nlm.nih.gov/geo/query/acc.cgi?acc=GSE78220. Additionally, well-recognized immune-related genes were downloaded from http://www.gsea-msigdb.org/gsea/msigdb/index.jsp (27).

Above datasets merely contain anonymized and de-identified patient information. Secondary analysis of de-identified data was confirmed exempt from review by the medical ethics committee of Hunan Cancer Hospital and the Affiliated Cancer Hospital of Xiangya School of Medicine as it was classified as negligible risk research. Thus, our study was exempt from the ethical review or the patient consent.

### 2.2 Establishment of a weighted gene co-expression network (WGCNA)

WGCNA is a widespread systematic algorithm used to generate gene modules with similar expression patterns and determine the correlations between modules and clinical traits (28). In this study, we screened the immune-related gene expression profiles in the IMmotion151 cohort and further identified a correlation network including significant clinical characteristics and genes using the “WGCNA” R package. We also developed an adjacency matrix to characterize the correlation strength between the nodes, which was further changed to a topological overlap matrix. Subsequently, modules containing more than 30 genes were identified *via* hierarchical clustering. To compare the co-expression levels, modules were clustered based on their correlation with module eigengenes (MEs). When the correlation of MEs > 0.80, module merging was performed, indicating that the expression profiles of the modules were similar (29). Pearson’s correlation coefficient was used to assess the correlations between the modules and various clinicopathological parameters. Finally, gene significance (GS) and module membership (MM) were used to quantify the relationships between the genes and the clinicopathological characteristics in the module. Hub genes were considered as those with MM > 0.8 and GS > 0.2 (29).

### 2.3 Identification of significant immune-related differentially expressed genes (DEGs) between responders and non-responders

Based on the results of WGCNA analysis, 269 immunotherapy-associated genes in the turquoise module were selected for further analysis. Meanwhile, DEGs between the patients with complete response (CR) and those with progressive disease (PD) in the IMmotion151 cohort were identified using the “ggplot2” R package (29). Nine immune-related genes significantly affecting the patient responsiveness to immunotherapy were ultimately identified using the intersection of the above two kinds of genes and used to construct an IRS model.

### 2.4 Functional and pathway enrichment analyses

Gene ontology (GO) and Kyoto Encyclopedia of Genes and Genomes (KEGG) analyses were conducted using the “clusterProfiler” R package to identify the DEG-associated signaling pathways and biological processes (30, 31). Pathways with a nominal P < 0.05 and false-discovery rate (FDR) < 0.05 were considered to be statistically significant.

### 2.5 Establishment and validation of an IRS model

Given the individual heterogeneity and intricacy of the clinical outcomes of advanced RCC cases treated with ICB combined with anti-angiogenic drugs, we formulated a scoring system, termed as the IRS model, using the ssGSEA algorithm on the basis of the mRNA expression levels of the identified nine immunotherapy-related genes in a single sample to quantify the prognostic level of each patients with mRCC for in-depth analysis.

Optimal cut-off point identified by the “surv-cutpoint” function of the “survminer” R package stratified all advanced RCC cases in the IMmotion151 cohort into high- or low-risk subgroups. In this approach, different values are grouped as cut-off values for statistical testing, and the result with the lowest P value is considered as the optimal cut-off point that corresponds to the most significant association with the clinical outcome.

A heat map was constructed to visualize the IRS distribution and clinicopathological parameters. Survival analysis between high- and low-risk group were conducted using Kaplan–Meier curves with log-rank test and the “survival” R package (32). Hazard ratios (HRs) and the corresponding 95% confidence intervals (CIs) were estimated.

Area under the curve (AUC) values of the receiver operating characteristic (ROC) curves established using the “survival ROC” R package were used to evaluate the predictive efficiency of the IRS model (33). In addition, to test the robustness of our IRS model, we verified its predictive capability using other external independent datasets: JAVELIN Renal 101, E-MTAB-3218, IMvigor210, and GSE78220 cohorts.

An identical median value based on the IMmotion151 cohort was also applied to the validation groups, effectively categorizing all patients into high- and low-risk groups.

### 2.6 Correlations between IRS and common biological processes

We further determined the correlations between IRS and subsequent biological processes. Mariathasan et al. (25) curated multiple gene sets associated with specific biological pathways, including (1) CD8^+^ T-effector signature (34), (2) antigen processing machinery (35), (3) epithelial–mesenchymal transition (EMT) biomarkers (26, 36, 37), (4) immune checkpoints (25).

### 2.7 Correlation between IRS and drug sensitivity

RNA-seq data of approximately 1000 tumor cell lines, AUC values for evaluating the efficacy of antineoplastic drugs in tumor cell lines, and targets or pathways of drugs were downloaded from the Genomics of Drug Sensitivity in Cancer (GDSC; https://www.cancerrxgene.org/) (38). Spearman correlation coefficient was used to evaluate the correlation between drug sensitivity and the IRS model, and |Rs| > 0.2 and P-value < 0.05 were considered to be significant.

## 3 Results

### 3.1 Use of WGCNA to screen immunotherapy-related genes

**Figure 1:**
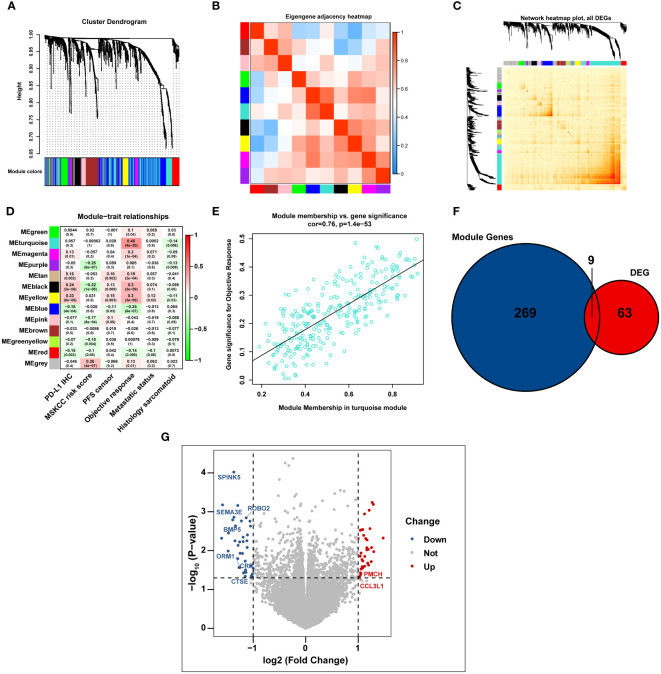
The immunotherapy-related genes are identified by WGCNA analysis. **(A)** Cluster dendrogram representing immune-related genes clustering based on different metrics. **(B, C)** Heatmap depicting the correlation coefficient in the modules. **(D)** Heatmap displaying the correlation between the module eigengenes and multiple clinical parameters in RCC. **(E)** Scatter plot exhibiting the correlation coefficient in the turquoise module. **(F)** Venn diagram illustrating the intersection of immune-related genes and immunotherapy-related DEGs in IMmotion150 cohort. **(G)** Volcano plot depicting immunotherapy-related DEGs between responders and non-responders.

All immune-related genes from GSEA are listed in **Supplementary Table 1**. After we intersected the above immune-related genes with the RNA-seq data of 407 patients with advanced RCC from the IMmotion151 cohort, 942 immune-related genes were identified and further subjected to WGCNA analysis (**Supplementary Table 2**). In line with the standard scale-free network distribution, the soft threshold power value was determined to be three (**Supplementary Figure 1**). Based on this dissimilarity, a dendrogram of all the gene clusters was formulated, which displayed 10 different modules (**Figure 1A**). Correlations among all clinical modules are illustrated in **Figures 1B, C**. We further assessed the correlations between MEs and clinical traits, including PD-L1 IHC, MSKCC risk score, PFS, objective response, metastatic status, and sarcomatoid histology. Turquoise module was most significantly associated with the objective response of patients with mRCC to atezolizumab plus bevacizumab (r = 0.45, P < 0.0001) (**Figure 1D**), indicating that genes in the turquoise module potentially exert a crucial effect on the clinical outcome of atezolizumab plus bevacizumab interventions. This turquoise module was further analyzed, and the genes in this module were found to be significantly correlated to the efficacy of atezolizumab plus bevacizumab therapy (r = 0.76, P < 0.0001) (**Figure 1E**).

We analyzed the gene expression profiles of 23 responders (patients with CR) and 75 non-responders (patients PD) in the IMmotion151 cohort, and identified 72 DEGs associated with the effects of atezolizumab plus bevacizumab (│log_2_FC│ > 1, P < 0.05, FDR < 0.05) (**Supplementary Table 3**). Moreover, we cross-referenced the above 72 DEGs and all genes in the turquoise module to select a total of nine DEGs, including seven downregulated DEGs in responders (serine protease inhibitor Kazal type 5 [*SPINK5*], semaphorin 3E [*SEMA3E*], roundabout guidance receptor 2 [*ROBO2*], bone morphogenetic protein 5 [*BMP5*], orosomucoid 1 [*ORM1*], C-reactive protein [*CRP*], and cathepsin E [*CTSE*]; log_2_FC < 1; P < 0.05) and two up-regulated DEGs in responders (promelanin-concentrating hormone (*PMCH*) and C-C motif chemokine ligand 3-like 1 [*CCL3L1*]; log_2_FC > 1; P < 0.05) (**Figures 1F, G**).

### 3.2 Biological functions of DEGs associated with the efficacy of atezolizumab plus bevacizumab therapy

**Figure 2:**
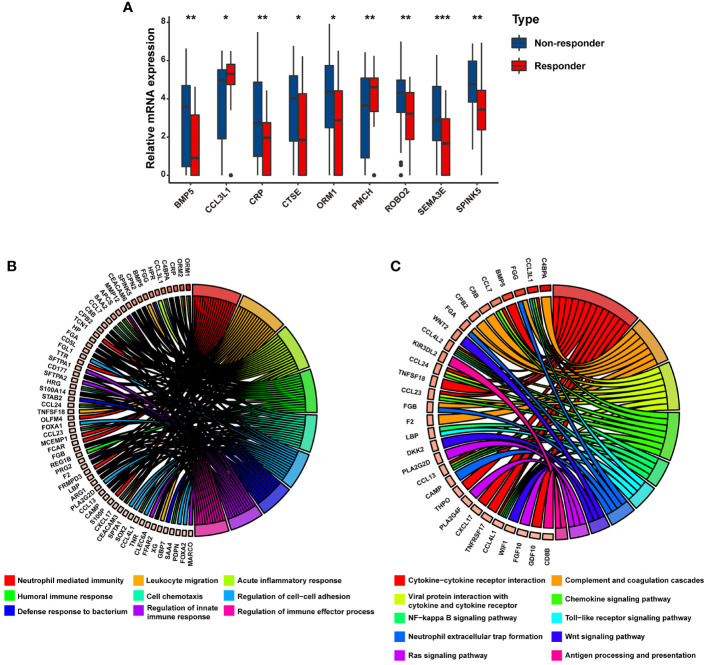
The potential biological processes associated with immunotherapy-related DEGs are determined by functional analysis. **(A)** Bar charts representing the expression levels of immunotherapy-related DEGs between responders and non-responders. Circular plot representing the potential biological pathways related to immunotherapy-related DEGs based on **(B)** GO analysis and **(C)** KEGG analysis. * p<0.05, ** p<0.01, *** p<0.001.

We further identified the mRNA expression profiles of the above nine genes in mRCC and found that, compared with the responders to atezolizumab plus bevacizumab therapy in the IMmotion151 cohort, the expression levels of seven genes (*SPINK5*, *SEMA3E*, *ROBO2*, *BMP5*, *ORM1*, *CRP*, and *CTSE*) were significantly increased and those of two genes (*PMCH* and *CCL3L1*) were decreased in the non-responders (P < 0.05) (**Figure 2A**). GO analysis of the nine DEGs linked them to neutrophil-mediated immunity, leukocyte migration, acute inflammatory response, humoral immune response, and cell chemotaxis (**Figure 2B** **;** **Supplementary Table 4**), most of which were associated with the modulation of immunity and immunotherapy. Similarly, KEGG pathway analysis revealed that these DEGs were correlated with cytokine–cytokine receptor interactions, complement and coagulation cascades, antigen processing and presentation, and neutrophil extracellular trap formation (**Figure 2C** **;** **Supplementary Table 5**), indicating their significance and conferring the basis to investigate a potential association between these genes and immunophenotypes.

### 3.3 Differences in the biological roles and clinical outcomes of IRS-high and -low groups

**Figure 3:**
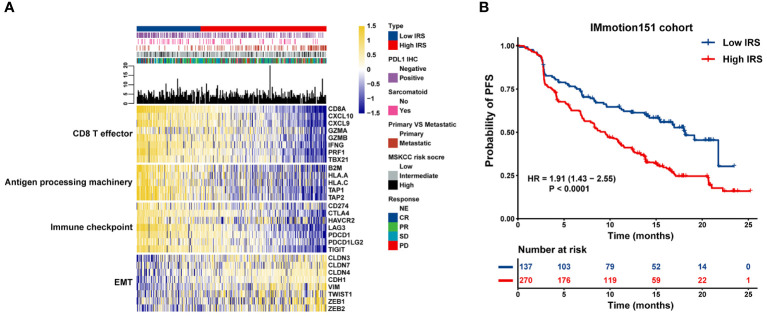
Association between transcriptional signatures, clinical outcome to atezolizumab plus bevacizumab and the IRS model. **(A)** Heatmap displaying the IRS distribution grouped by transcriptional signatures. **(B)** Kaplan-Meier curves of PFS in mRCC patients with high or low IRS in the IMmotion151 cohort.

Based on the strength of the optimal cut-off point (–0.46), 407 individuals with mRCC were stratified into high- and low-risk groups (high: IRS > –0.46 and low: IRS < –0.46) (**Supplementary Table 6**). To investigate the potential mechanism of the impact of IRS on atezolizumab plus bevacizumab therapy for mRCC, a combined heat map was constructed to visually demonstrate the correlations between IRS and multiple clinicopathological parameters, including PD-L1 IHC, MSKCC risk score, objective response, metastatic status, and sarcomatoid histology, in the high and low IRS groups. We compared the high and low IRS groups in the IMmotion151 cohort and found that more patients in the IRS-low group had positive PD-L1 IHC results than those in the IRS-high group. Metastatic tumors were primarily distributed in the IRS-high cluster, whereas the proportion of sarcomatoid histological subtypes in the IRS-low group was greater than that in the IRS-high group. Additionally, patients with low IRS were primarily characterized by a higher expression of CD8^+^ T-effectors, antigen-processing machinery, and immune checkpoint signatures in the IRS-low group than in the IRS-high group. Conversely, patients in the IRS-high group showed relatively high expression levels of EMT-associated genes (**Figure 3A**). We further assessed the clinical outcomes of patients treated with atezolizumab plus bevacizumab in the IRS-high and -low groups. Survival analysis revealed that short PFS in patients with high IRS (HR = 1.91; 95% CI = 1.43–2.55; P < 0.0001) (**Figure 3B**).

**Figure 4:**
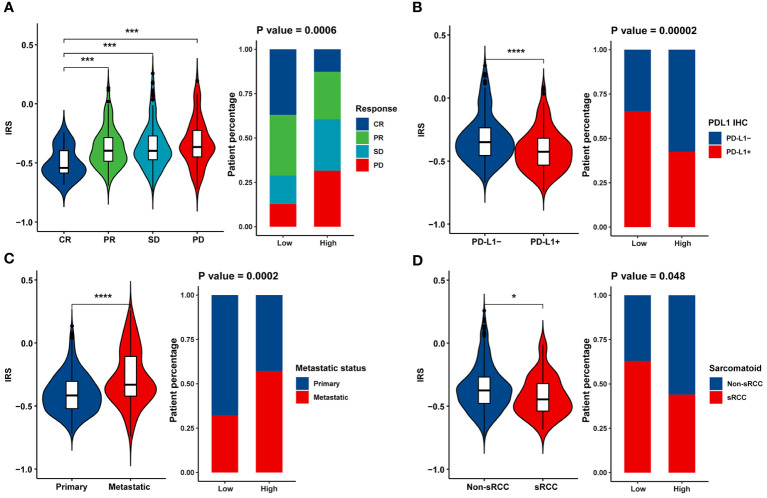
Association between clinicopathological parameters and the IRS model. Violin plot and bar chart howing the correlation between the IRS and **(A)** response to Atezolizumab + Bevacizumab, **(B)** PD-L1 expression, **(C)** metastatic status and **(D)** histological subtype. * p<0.05, *** p<0.001, **** p<0.0001.

Furthermore, IRS was significantly higher in the SD/PD group than in the CR/PR group (**Figure 4A**), indicating that IRS was negatively associated with the magnitude of response to atezolizumab plus bevacizumab in mRCC. Compared to tumors that were negative for PD-L1 IHC, tumors that were positive for PD-L1 IHC exhibited lower IRS (**Figure 4B**). We also observed enrichment of metastatic tumors (**Figure 4C**) and sarcomatoid histological subtypes (**Figure 4D**) in the IRS-high group. These findings suggest that the IRS can predict the efficacy of atezolizumab plus bevacizumab.

### 3.4 Validation of the IRS model with multiple immunotherapy datasets

**Figure 5:**
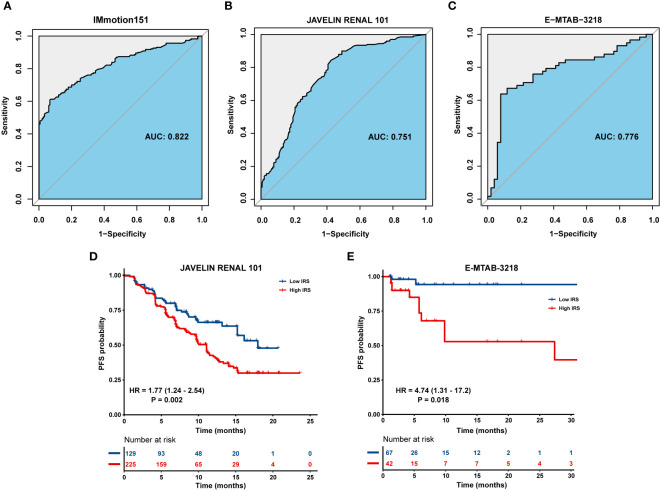
Validation of the IRS in multiple immunotherapy datasets. ROC curve displaying the predictive power of the IRS in **(A)** IMmotion151 cohort, **(B)** JAVELIN Renal 101 cohort and **(C)** E-MTAB-3218 cohort. Kaplan-Meier curves of PFS in tumor patients with high or low IRS in **(D)** JAVELIN Renal 101 cohort and **(E)** E-MTAB-3218 cohort.

ROC curve was used to determine the ability of the IRS model to distinguish between immunotherapy responders and non-responders. The IRS model displayed a satisfactory performance to differentiate responders from non-responders, with an AUC of 0.822 (95% CI = 0.782–0.863) in the IMmotion151 cohort (**Figure 5A**). We also selected two external independent datasets: the JAVELIN Renal 101 cohort (patients with mRCC treated with a combination of avelumab and axitinib) and the E-MTAB-3218 cohort (patients with mRCC treated with nivolumab). When assessing survival prediction, we found that the AUC of our IRS model was 0.751 (95% CI = 0.699–0.803) in the JAVELIN Renal 101 cohort (**Figure 5B**) and 0.776 (95% CI = 0.684–0.868) in the E-MTAB-3218 cohort (**Figure 5C**). Additionally, we validated our model in the IMvigor210 cohort (patients with advanced urothelial cancer who received atezolizumab therapy) and GSE78220 (patients with metastatic melanoma who received pembrolizumab therapy), with AUC of 0.902 (95% CI = 0.868–0.936) (**Supplementary Figure 2A**) and 0.879 (95% CI = 0.7437–1) (**Supplementary Figure 2B**), respectively. Therefore, our results highlight that IRS has a favorable capability to stratify a subset of patients who will benefit from immunotherapy.

Patients with high IRS exhibited an inferior prognosis compared to those with low IRS in the JAVELIN Renal 101 cohort (HR = 1.77; 95% CI = 1.24–2.54; P = 0.002) (**Figure 5D**) and E-MTAB-3218 cohort (HR = 4.74; 95% CI = 1.31–17.2; P = 0.018) (**Figure 5E**). Similarly, patients in IRS-high group were characterized with a shorter OS than those in IRS-low group in the IMvigor210 cohort (HR = 2.59; 95% CI = 1.87–3.58; P < 0.001) (**Supplementary Figure 2C**) and GSE78220 cohort (HR = 4.22; 95% CI = 1.11–16.0; P = 0.034) (**Supplementary Figure 2D**).

### 3.5 Correlation between IRS and anti-tumor chemotherapy efficacy

**Figure 6:**
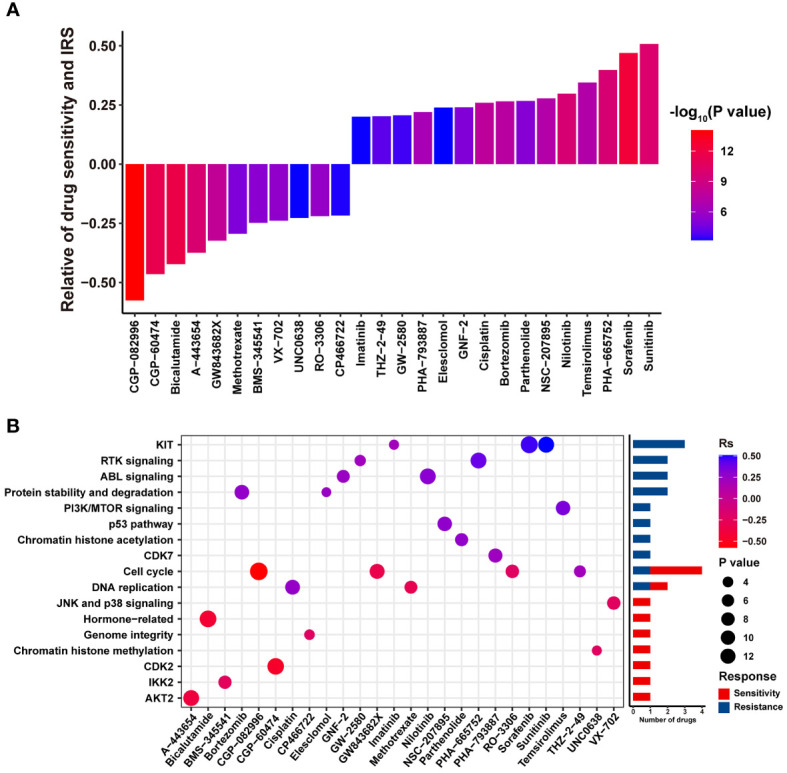
The potential relationship between the IRS model and efficacy of antitumor chemotherapy. **(A)** Box diagram displaying the correlation between the IRS and drug sensitivity. Rs > 0 or Rs < 0 indicated drug resistance or drug sensitivity, respectively. **(B)** Dot plot summarizing the signal pathways related to drugs that were resistant or sensitive to the IRS.

A total of 26 correlated pairs between the IRS model and drug sensitivity in the GDSC database were analyzed using Spearman’s correlation analysis (38). There was significant correlation between drug sensitivity and IRS in 11 pairs, including CGP-082996, CGP-60474, and bicalutamide (Rs < –0.2, P < 0.05). In contrast, 15 pairs, including sunitinib, sorafenib, and temsirolimus (Rs > 0.2, P < 0.05), were characterized by significant correlation between drug resistance and the IRS model (**Figure 6A**). In addition, drugs whose sensitivity correlated with low IRS primarily targeted chromatin histone acetylation, *p53* pathway, phosphoinositide 3-kinase (*PI3K*)*/*mammalian target of rapamycin (*MTOR*) signaling and protein stability, and degradation signaling pathways. However, drugs whose sensitivity was linked to high IRS mostly targeted the *AKT2*, *IKK2*, *CDK2*, and chromatin histone methylation signaling pathways (**Figure 6B**). Collectively, these results indicate that our IRS model is also associated with chemotherapy response in RCC.

## 4 Discussion

In our study, specific promising gene biomarkers were determined by investigating RNA-Seq data from the IMmotion151 cohort. Currently, The Cancer Genome Atlas (TCGA) and Gene Expression Omnibus (GEO) databases are considered as data sources for developing RCC prognosis prediction models in the majority of publications, which fails to effectively promote the prediction accuracy of immunotherapy in RCC. Thus, we extracted mRCC cases from the IMmotion151 cohort to conduct this study, which avoided the potential effect of non-locally advanced RCC on risk prediction models and scoring systems.

We established an immune-related risk score model to evaluate the efficacy of atezolizumab plus bevacizumab in patients with mRCC and further verified our model based on multiple cohorts. Our report demonstrated that in the IMmotion151 cohort, mRCC cases with low IRS were associated with a favorable prognosis and effective responsiveness to atezolizumab plus bevacizumab. Hub genes significantly associated with the efficacy of atezolizumab plus bevacizumab in the turquoise module were initially identified using WGCNA. Nine immunotherapy-related DEGs were confirmed following the overlap of hub genes and DEGs between patients with PD and CR.

Among the nine immunotherapy-related DEGs (*SPINK5*, *SEMA3E*, *ROBO2*, *BMP5*, *ORM1*, *CRP*, *CTSE*, *PMCH*, and *CCL3L1*), differential analysis showed that *SPINK5* was significantly overexpressed in head and neck squamous cell carcinoma (HNSCC) samples compared to that in normal tissues, and *SPINK5* expression levels were positively associated with Treg cells in the tumor microenvironment (39). *SEMA3E* triggered macrophages-mediated inflammation (40). Inflammation contributes greatly to tumorigenesis and tumor development (41), indicating that *SEMA3E* potentially accelerates tumor progression by regulating chronic inflammation, which is a hallmark of various malignancies (42). ROBO2 belongs to the ROBO family and is a conserved transmembrane receptor protein that is primarily located in the nervous system, vascular endothelial cells, and muscle cells (43). *SLIT2/ROBO2*-mediated PI3K-γ activation accelerated microglia/macrophage chemotaxis and tumor-supportive polarization, thus enhancing macrophage invasion and diminishing efficacy of chemotherapy and immunotherapy in gliomas (44). BMP5 is recognized as a secreted growth factor and a member of the transforming growth factor-beta superfamily, which exerts crucial effects on the pathogenesis of inflammatory and autoimmune disorders, including Keshan disease (45) and autoimmune encephalomyelitis (46), *BMP5* triggered keratin expression in adherent bone marrow cells, thereby contributing to the progression of chronic cutaneous neoplasms (47). ORM1 is linked to tumor immunity, including antigen processing and presentation, T-cell receptor signaling, and cytokine-cytokine receptor interactions. Specifically, ORM1 potentially acts as an inhibitory factor to protect tumor cells from attack by the immune system, thereby leading to the immune escape of tumors (48). CRP is a biomarker of systemic inflammation and can be generated by RCC cells (49). Increased CRP levels are linked to the infiltration of immunosuppressive cells, including regulatory T (Treg) cells and tumor-associated macrophages, and thus predict undesirable outcomes in patients (50–52). CTSE is associated with lipid metabolism. CTSE participates in antigen processing and modulates the processing of antigenic peptides during MHC class II-mediated antigen presentation (53). Some studies have demonstrated that *CTSE* is overexpressed in tumor tissues than in normal tissues in various types of cancer, such as bladder cancer (53), pancreatic cancer (54), and hepatocellular carcinoma (55). *PMCH* functions as a neuromodulator of neuronal function that regulates goal-directed behavior (56). Downregulation of *PMCH* in ccRCC is significantly associated with advanced TNM stage, distant metastasis, and undesirable outcomes (57). CCL3L1 belongs to the CC chemokine family, which exerts an anti-tumor effect by inducing multiple immune cells, including CD8+ T cells and immature dendritic cells (58). However, *CCL3L1* overexpression is also involved in the progression of glioblastoma (59). Therefore, the characteristics of *CCL3L1* in RCC should be further explored. Thus, the correlation between certain immunotherapy-related DEGs and immunotherapy may provide promising targets for immune checkpoint inhibitors in RCC treatment.

Notably, these genes were primarily associated with inflammation and immune responses. Inflammation is a well-known hallmark of tumor progression. Various inflammatory signaling cascades are closely related to tumorigenesis and the development of RCC, particularly the *VHL* (60), *mTOR*, tumor necrosis factor (*TNF*), and signal transducer and activator of transcription pathways (60–63). Additionally, inflammation-associated factors, TNF-α, CXCR4 and CCR3, are significantly correlated with the prognosis and staging of RCC cases (64). Inhibition of pro-inflammatory pathways may be an effective strategy to retard the development of RCC. For example, LY294002, which targets the PI3K/AKT pathway, is potentially conducive to the prognosis of patients with RCC (65). Immunotherapy with nivolumab combined with ipilimumab has great potential for the treatment of RCC (66).

It has been demonstrated that kidney stone disease (KSD) is linked to RCC and RCC is more frequent among individuals with kidney stones (67–70). KSD is primarily composed of monohydrate (COM) crystals (71). COM crystals triggers renal cell injury through inducing reactive oxygen species overproduction and accelerating oxidative DNA damage (71, 72). Oxidative DNA damage exerts crucial effects on inflammation and the initiation and development of RCC (73, 74).

A recent study demonstrated that COM crystals accelerate the process of EMT, strengthen the invasion ability, cell-aggregate formation, chemoresistance to cisplatin, and secretion of VEGF, and trigger the overexpression of oncogene *TPX2* and the downregulation of tumor suppressor genes, including *PTEN*, *VHL*, and *ARID1A*, which are conventional inflammation-associated factors, ultimately exhibiting several carcinogenic characteristics in non-cancerous renal cells (70). Thus, there is a potential and sophisticated crosstalk between KSD, RCC, and inflammation.

The validation in additional four dependent cohorts demonstrated that the risk model exhibited satisfactory and robust prediction efficiency. The diagnosis and treatment of individuals with tumors will benefit from the validity and rationality of constructing a model based on big data algorithms. A risk model incorporating nine genes has generally been studied for multiple tumors; however, there are no reports on immunotherapy-related risk models for RCC. Patients with mRCC with low-risk scores showed improved PFS and could benefit from the dual combination of nivolumab plus ipilimumab, as evaluated by the IRS model, whereas cases in the high-risk group displayed numerically inferior results for PFS with nivolumab + ipilimumab. In this study, patients with mRCC with low risk scores were enriched in the CD8^+^ T effector, antigen processing machinery, and immune checkpoint pathways. In contrast, patients with high-risk scores displayed greater expression of EMT-related genes. These results provide a molecular explanation for the better prognosis of favorable-risk cases with therapeutic regimens comprising nivolumab + ipilimumab. Previous studies have shown that ICIs block inhibitory immune receptors and activate dysfunctional T cells, including CD8^+^ T cells. CD8^+^ T effectors in the adaptive immune system exert a potent anti-tumor immune response and form the cornerstone of tumor immunotherapy (75). The antigen-processing machinery exerts a vital impact on the synthesis and expression of HLA class I tumor antigen-derived peptide complexes that trigger the identification and elimination of malignant cells mediated *via* cognate T cells (76).

We performed a thorough molecular analysis of 407 samples from patients with advanced RCC who underwent atezolizumab plus bevacizumab therapy and further established the first prognostic model to accurately distinguish responders from non-responders based on a randomized global Phase III clinical trial IMmotion151 cohort. Specifically, patient-reported outcomes (PROs) in IMmotion151 suggest a lower overall treatment burden with atezolizumab plus bevacizumab than with sunitinib in patients with treatment-naïve mRCC and provide further evidence for the clinical benefit of this regimen. A report evaluated PROs in the phase III IMmotion151 trial and demonstrated that compared with sunitinib in patients with mRCC, those receiving atezolizumab plus bevacizumab therapy were characterized by a lower overall therapy burden, including longitudinal and time to deterioration for core and RCC symptoms and their interference with daily life, therapy side effects, and health-related quality of life (77). Another study indicated that although a clinical benefit was revealed in atezolizumab plus bevacizumab based on PFS analysis, the final analysis exhibited a similar median OS in patients treated with atezolizumab plus bevacizumab and sunitinib. Biomarker analysis demonstrated that sunitinib improved the median OS in patients whose tumors were characterized by a higher prevalence of angiogenesis; conversely, atezolizumab plus bevacizumab displayed a trend of improved OS in tumors with poor angiogenesis, but T-effector/proliferative, proliferative, or small nucleolar RNA transcriptomic profiles. These results potentially provide guidance for the individualized treatment of patients with mRCC (78).

This study has some limitations. Owing to the limited number of patients receiving immunotherapy and the complexity and difficulty in collecting clinical tissues from patients with advanced RCC treated with immunotherapy, we failed to conduct external verification based on our own dataset. Nevertheless, we validated our IRS model using four additional public immunotherapy cohorts to overcome this disadvantage. Moreover, our IRS model comprised nine immunotherapy-related DEGs. The biological properties and potential molecular mechanisms of these genes in mRCC need to be explored to facilitate the widespread clinical application of IRS models.

## 5 Conclusion

In conclusion, we identified the most significant immunotherapy-associated genes in patients with advanced RCC from the IMmotion151 cohort and developed a novel and promising immunotherapy prediction model and scoring system to estimate the responsiveness of patients with advanced RCC to atezolizumab plus bevacizumab therapy. Our model can further aid in patient stratification and development of personalized therapies for patients with untreated advanced RCC.

## Data availability statement

The original contributions presented in the study are included in the article/**Supplementary Material**. Further inquiries can be directed to the corresponding author.

## Ethics statement

Five datasets analyzed in our report merely contain anonymized and de-identified patient information. Secondary analysis of de-identified data was confirmed exempt from review by the medical ethics committee of Hunan Cancer Hospital and the Affiliated Cancer Hospital of Xiangya School of Medicine as it was classified as negligible risk research. Thus, our study was exempt from the ethical review or the patient consent.

## Author contributions

XY and PC designed/planned the study and wrote the paper. PC performed computational modeling, acquired, and analyzed data. PC, FB, WT, and LJ performed imaging analysis. PC, FB, WT, LJ, and XY participated in discussion of related data. XY and PC drafted the manuscript. All authors contributed to the article and approved the submitted version.

## Supplementary material

Supplementary Figure 1 The scale-free fit index and the mean connectivity of the soft-thresholding powers.

Supplementary Figure 2 Validation of the IRS in the IMvigor210 cohort and GSE78220. ROC curve displaying the predictive power of the IRS in (A) IMvigor210 cohort, (B) and GSE78220 cohort. Kaplan-Meier curves of OS in tumor patients with high or low IRS in (C) IMvigor210 cohort and (D) GSE78220 cohort.
