# Supplementary figures and images for: A novel immune-related model to predict prognosis and responsiveness to checkpoint and angiogenesis blockade therapy in advanced renal cancer
Source: Front Oncol. 2023 Mar 14;13:1127448. doi: 10.3389/fonc.2023.1127448 (PMC10043594; doi:10.3389/fonc.2023.1127448)

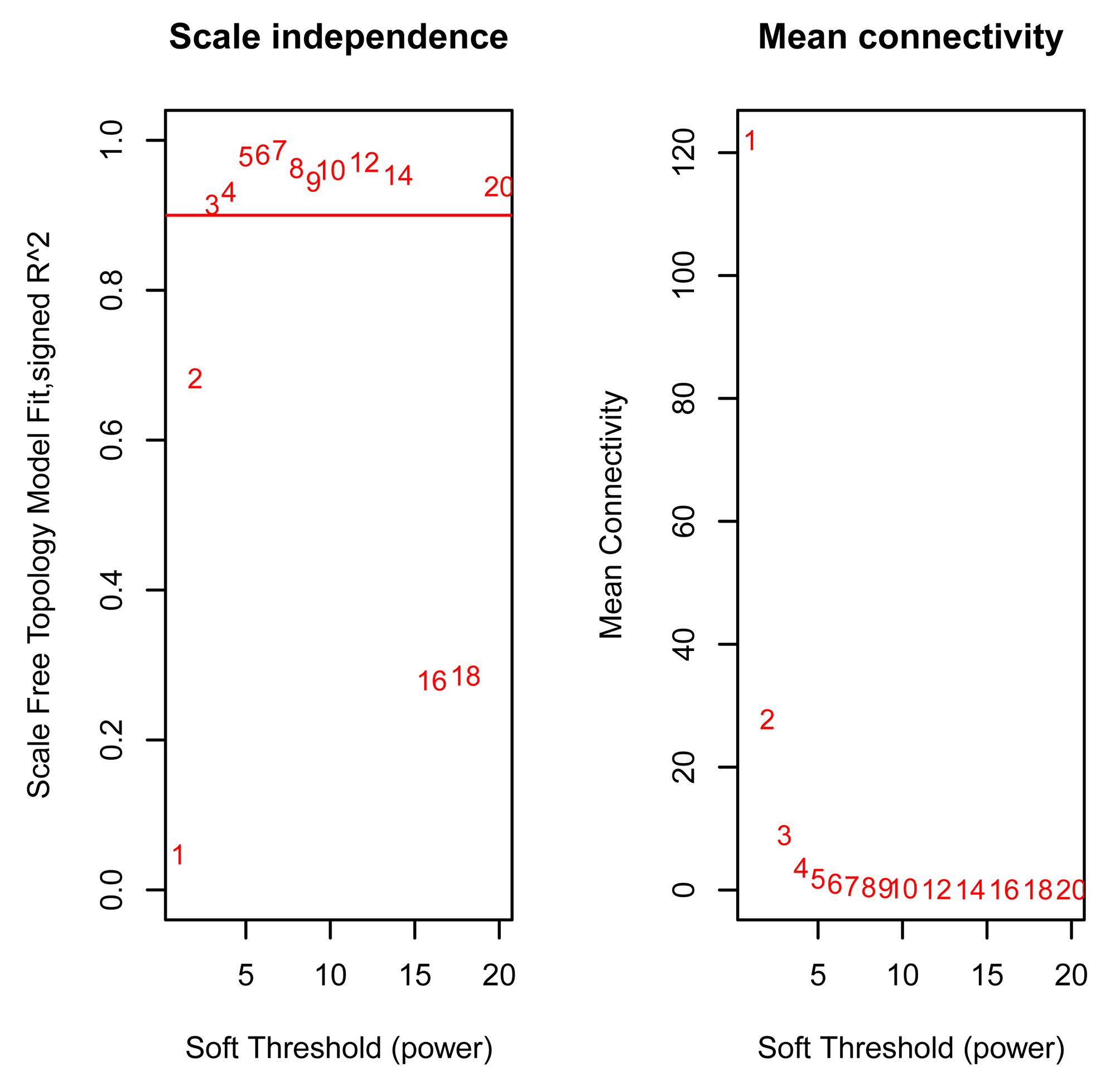

Supplement: Supplementary Figure 1 — The scale-free fit index and the mean connectivity of the soft-thresholding powers. [file Image_1.tif]

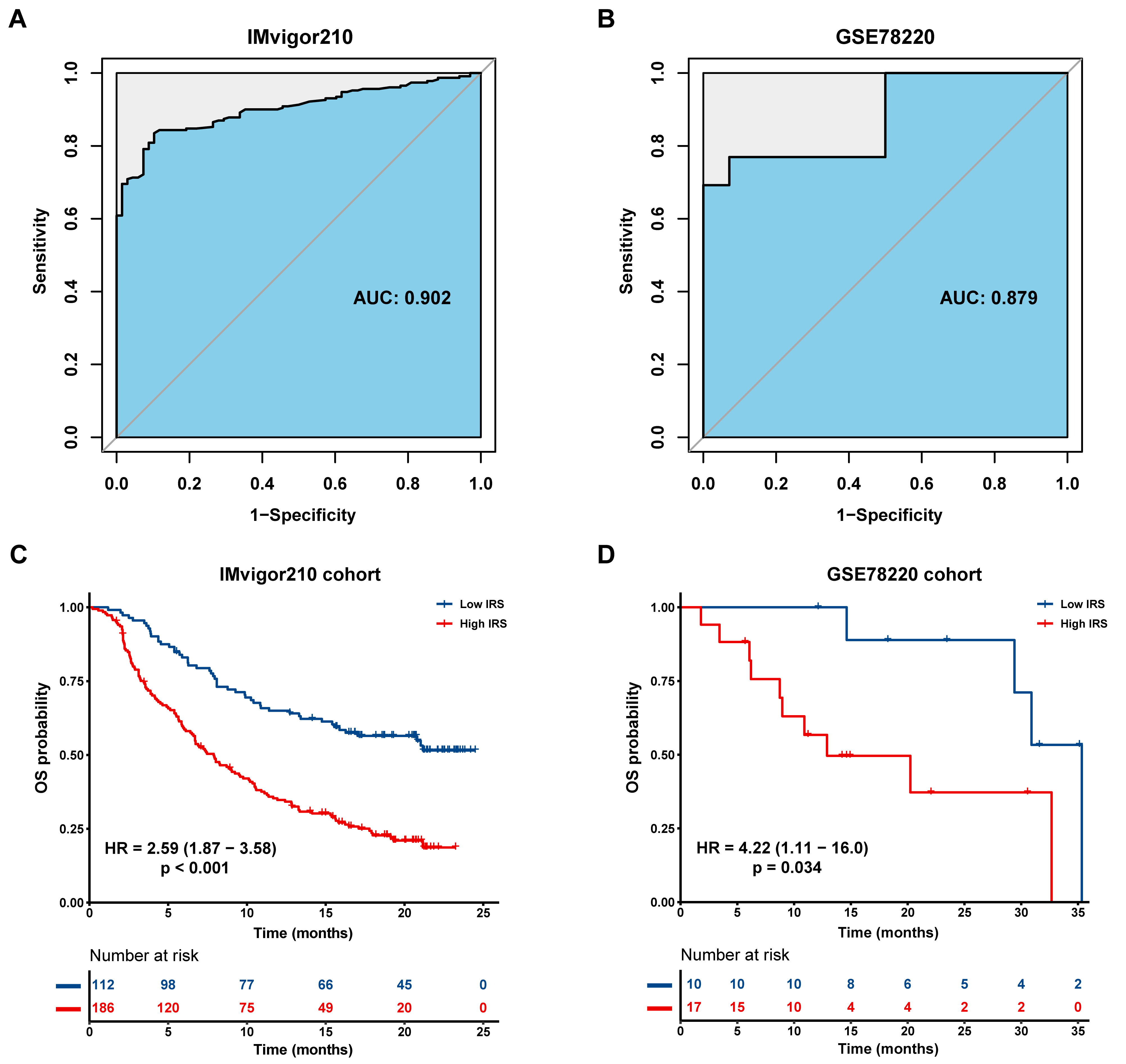

Supplement: Supplementary Figure 2 — Validation of the IRS in the IMvigor210 cohort and GSE78220. ROC curve displaying the predictive power of the IRS in (A) IMvigor210 cohort, (B) and GSE78220 cohort. Kaplan-Meier curves of OS in tumor patients with high or low IRS in (C) IMvigor210 cohort and (D) GSE78220 cohort. [file Image_2.tif]
